# Exploration of phase structure evolution induced by alloying elements in Ti alloys via a chemical-short-range-order cluster model

**DOI:** 10.1038/s41598-019-40302-5

**Published:** 2019-03-04

**Authors:** Beibei Jiang, Qing Wang, Chuang Dong, Peter K. Liaw

**Affiliations:** 1 Key Laboratory for Materials Modification by Laser, Ion and Electron Beams (Ministry of Education), School of Materials Science and Engineering, Dalian University of Technology, Dalian, 116024 China; 2 Department of Materials Science and Engineering, The University of Tennessee, Knoxville, Tennessee 37996 USA

## Abstract

The prominent comprehensive properties of solid-solution- and intermetallic-based Ti alloys are derived from their diverse microstructures induced by multi-component alloying, which results in a chemical composition complexity. A cluster-plus-glue-atom model, characterizing the chemical short-range orders, was introduced to explore the relationships among the local atomic distributions of alloying elements in different phase structures of Ti alloys, including *α*-Ti, *β*-Ti, *ω*-Ti, *α*_2_-Ti_3_Al, *γ*-TiAl, *O-*Ti_2_AlNb, and B2-Ti(Al,Nb). Specific cluster structural units, *i*.*e*., cluster formulas, for these phases were determined with the guide of the Friedel oscillation theory for electron-structure stabilization. It is due to the change of cluster structural units that induces the phase transformation, which is attributed to the amounts of primary alloying elements of Al and Nb. The total atom number (*Z*) values in these cluster structural units, calculated by the Fermi vector *k*_F_, are all very close to the integer of *Z* = 16. Furthermore, the composition rules of industrial multi-component Ti alloys based on these phases were generalized in light of the cluster formula approach, which will open up a new route towards designing high-performance Ti alloys with complex compositions.

## Introduction

Titanium alloys have been used extensively in many industrial fields due to their high specific strength, excellent high-temperature oxidation and corrosion resistances, as well as good capacity for preventing crack propagation^1–3^. They are primarily alloyed by the Al element and generally classified into two groups, solid-solution-based and intermetallic-based alloys. Solid-solution-based Ti alloys with a lower amount of Al, including *α*-Ti (a hexagonal close-packed (HCP) structure), (*α* + *β*)-Ti, and *β*-Ti (a body-centered cubic (BCC) structure) alloys, have the widest applications in aerospace, shipbuilding and chemical industry below the temperature of 650 °C^4,5^. Intermetallic-based light-weight Ti alloys with a higher amount of Al, mainly containing *α*_2_-Ti_3_Al (D0_19_ *hP*-Ni_3_Sn type), *O-*Ti_2_AlNb (*oC*-HgNa type), or *γ*-TiAl (L1_0_ *tP*-AuCu type) based alloys, are widely used in the aerospace industry due to their excellent high-temperature oxidation- and creep-resistant properties^6–8^. In order to ensure the structural stability and to improve further their performance, multiple alloying elements (Mo, Nb, V, Cr, Zr, Si, etc.) were often co-added into these Ti alloys, such as the near *α*-IMI834 (Ti-5.8Al-4Sn-3.5Zr-0.7Nb-0.5Mo-0.35Si-0.06 C, wt.%)^9^, near *β*-Ti55531 (Ti-5Al-5Mo-5V-3Cr-1Zr, wt.%)^10^, Ti_3_Al-based Ti-25Al-10Nb-3V-1Mo (at.%)^11^, and TiAl-based Ti-43Al-4Nb-1.5Mo-0.1B (at.%)^12^ alloys.

Intrinsically, the superior mechanical properties of different types of Ti alloys are dependent on their diverse microstructures, which could be affected by alloying element species and their contents on the premise of fixing processing^4^. Thereof, some practical methods, such as Al- and Mo- equivalent methods^13–15^, *d*-electron method^16^, and first-principles calculation^17^, have been put forward to design multi-component Ti alloys efficiently. In our previous work, we proposed a ‘cluster-plus-glue-atom’ structural model to describe the local atomic distribution of alloying elements based on the chemical short-range orders (CSROs)^18–23^. CSRO is the most typical structural characteristic of solid solutions due to the obvious local structural heterogeneities with respect to the average crystal structure, which plays an important role to the various mechanical and physical properties of alloys^24–28^. In this cluster model, the cluster is the nearest-neighbor polyhedron centered by a solute atom who has the strong interaction with the base solvent atoms to represent the strongest CSRO. Some other solute atoms (*i*.*e*., glue atoms) with weak interactions are certainly required to fill the space between the clusters to balance the atomic-packing density. Thus, a uniform composition formula [cluster](glue atom)_*x*_ (*x* being the glue atom number) of the cluster structural unit, can be obtained from the cluster model, named the cluster formula approach^20,23^. Then, the sites of solute elements in the cluster formula is determined according to the enthalpy of the mixing (Δ*H*)^29^ between elements, which can represent the interaction between the solute and the base element. That is to say, the solute element with a large negative Δ*H* preferentially occupies the cluster center site to form a stable cluster, while that with a positive Δ*H* tends to occupy the glue atom site. Besides, the cluster-shell sites are primarily occupied by the base solvent atoms, as well as some solutes having a zero Δ*H* with the base. So the universal cluster formula [cluster](glue atom)_*x*_, containing all the key information on the alloy chemistry, *i*.*e*., chemical compositions, atomic occupancies (at the center, shell and glue sites) and chemical bonds in a cluster structural unit, can be regarded as the molecular formula for alloy structure. In fact, neutron-scattering experiments have demonstrated that the local atomic distribution of solutes in solid solutions is consistent with that in intermetallics, which is closely related to the atomic interactions^30,31^. This cluster formula approach has been successfully used to design a series of multi-component *β*-Ti alloys with low Young’s moduli, such as *β*-[(Mo_0.5_Sn_0.5_)-(Ti_13_Zr_1_)]Nb_1_ with a Young’s modulus of 48 GPa on the premise of the BCC structural stability^32,33^. There exist six common phase structures in Ti solid-solution- and intermetallic-based alloys, being the HCP *α*-Ti, BCC *β*-Ti, HCP *α*_2_-Ti_3_Al (the ordered superstructure of *α*-Ti), tetragonal *γ*-TiAl, orthorhombic *O-*Ti_2_AlNb, and B2 (*cP*-CsCl type, the ordered superstructure of *β*-Ti), respectively. With increasing the amounts of alloying elements, the ordered phases will certainly precipitate from their parent solid solutions, such as *α*_2_-Ti_3_Al from its parent *α*-Ti solid solution, in which the atomic distribution of Al solutes transforms from the short-range order to long-range order^34,35^. Therefore, from this viewpoint of chemistry, the present work will explore the relationships among the local atomic distributions of alloying elements in these phases via the cluster-plus-glue-atom model for the characterization of CSROs. The specific cluster structural units, *i*.*e*., cluster formulas, for these phases will be then determined with the guide of the Friedel oscillation theory for the electron-structure stabilization^36^. Finally, the composition rules of industrial multi-component Ti alloys based on these phases will be generalized in light of the cluster formula approach, which will provide a theoretical guidance for designing complex compositions of high-performance Ti alloys.

## Local Atomic Distributions of Alloying Elements in Ti-Al(-Nb) Phases

It is known that almost all of industrial Ti alloys were developed on the basis of the Ti-Al binary system. In the Ti-Al binary phase diagram^37^, the high-temperature BCC *β*-Ti solid solution covers a wide composition range, reaching up to 44.8 at.% Al, while only an amount of 12.5 at.% Al could dissolve in the HCP *α*-Ti at low temperatures. The transformation of BCC-*β* → HCP-*α* is induced by the decrease of the free energy with temperature, in which the solubility of Al is gradually reduced. Above this content of 12.5 at.%, the ordered *α*_2_-Ti_3_Al phase will precipitate from its parent *α*-Ti solid solution, resulting in a precipitation strengthening of the matrix. When the Al content falls into the range of 20 ~ 35.5 at.%, alloys generally possess a single *α*_2_ structure and have an excellent high-temperature stability. The tetragonal *γ*-TiAl phase with a L1_0_-CuAu structure forms with the Al content above 50 at.%, which can be stabilized till the melting temperature (~1450 °C). It was found that the addition of the BCC-stabilizer Nb into both *α*_2_-Ti_3_Al and *γ*-TiAl can contribute to the formation of the orthorhombic *O*-Ti_2_AlNb or ordered B2-Ti(Al,Nb) phases^38–40^. Besides, the additions of β stabilizers into *β*-Ti can restrain the transformation of *β*-Ti → *α*-Ti, which favors to the formation of the intermediate phase of *ω*-Ti (*hP*-AlB_2_ type).

**Figure 1:**
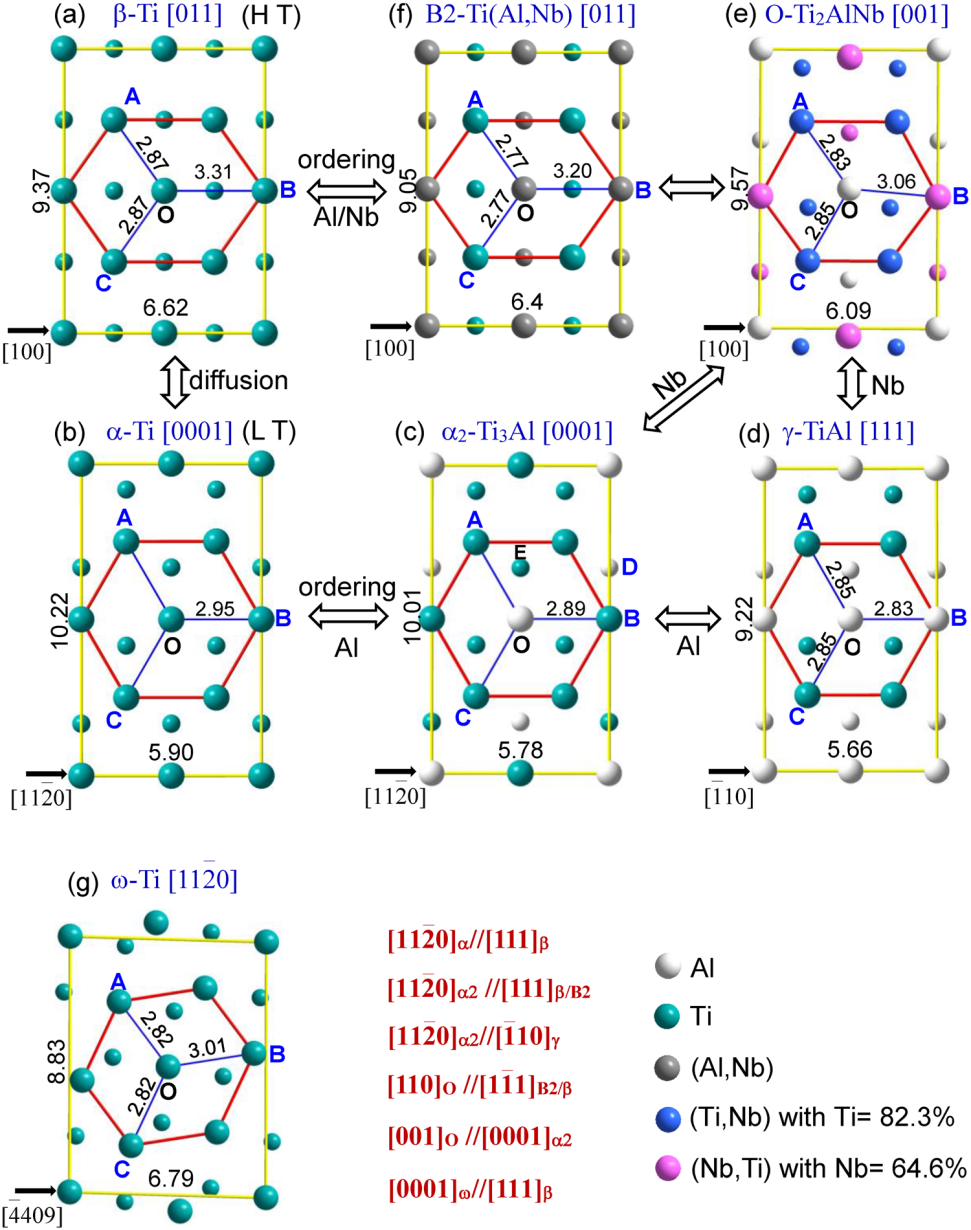
Local atomic distributions of *β*-Ti, *ω*-Ti, *α*-Ti, *α*_2_-Ti_3_Al, *O*-Ti_2_AlNb, *γ*-TiAl, and B2-Ti(Al,Nb) phases in the close-packed [110], [11$\bar{2}$ 0], [0001], [0001], [001], [111] and [110] projections, respectively, as well as their orientation relationships among these phases. The small atom symbols denote the atomic positions c/2 below the large ones. The lengths in Å of AB, AC, BC, and the rectangle sides are also shown between the two adjacent atoms. The ‘L T’ and ‘H T’ are abbreviated words of low and high temperature, respectively.

Figure 1 shows the local atomic distributions of *β*-Ti, *ω*-Ti, *α*-Ti, *α*_2_-Ti_3_Al, *O*-Ti_2_AlNb, *γ*-TiAl, and B2-Ti(Al,Nb) phases in the close-packed [110], [11$\bar{2}$0], [0001], [0001], [001], [111], and [110] projections, respectively. Obviously, there exist some small adjustments in the distribution of the solute atoms (Al and Nb) among these phases, although all the nearest-neighbor atomic distributions surrounding the center atom (O point) show a hexagonal shape in their close-packed planes. Specifically, the distances between the center O and the hexagonal vertices (A, B, and C points) are not equal in these phases, as seen in Fig. 1. The distance should become shorter gradually with the addition of Al into *α*-Ti due to the strong interaction between Al and Ti, as exemplified by the length of the OB segment (Ti-Ti bond) changing from 2.95 Å in pure *α*-Ti to 2.89 Å (Al-Ti bond) in *α*_2_-Ti_3_Al (Fig. 1(b,c)). It is noted that the nearest-neighbor shell sites (the hexagonal vertices) of the center Al in *α*_2_ is still surrounded by solvent Ti atoms, same as that in *α*-Ti solid solution. However, the second nearest-neighbor shell sites, like the D site located at the c/2 layer, are occupied by the Al atoms to form the ordered phase *α*_2_-Ti_3_Al of *α*-Ti (Fig. 1(c)). With further increasing Al content, Al atoms will enter into the nearest-neighbor shell sites, besides the center Al, which certainly leads to further shorten the OA, OB, and OC lengths and results in a small distortion in the hexagon (Fig. 1(d)). This tendency will be obvious when the Nb atoms, rather than the Al, enter into the nearest-neighbor shell in the *O*-Ti_2_AlNb phase (Fig. 1(e)). Thus, the hexagon will be distorted seriously and Nb atoms tend to separate the nearest-neighbor shell into two shells, as exemplified by the atomic distributions in B2 and BCC phases (Fig. 1(a,f)). Simultaneously, the atoms located at the c/2 layer shift up till to be aligned with the atom A, as seen the evolution from *α*_2_ to B2 or BCC (Fig. 1(a,c,f)). For the intermediate phase *ω*-Ti between *β*-Ti and *α*-Ti, the nearest-neighbor hexagon seems to rotate anticlockwise (Fig. 1(g)), compared with that in *β*-Ti. From this viewpoint, the local atomic distribution in *ω*-Ti is much closer to that in *β*-Ti. The structure information of all these phases, including lattice constants and atomic distributions in several nearest-neighbor shells are listed in the Supplementary Table S1. Since there exist specific CSROs in different structures from the close-packed projections (Fig. 1), we will explore the relationships of the local atomic distributions of solutes among these phase via the special cluster-plus-glue-atom model that characterizes the CSROs in the following.

## Cluster-Plus-Glue-Atom Model and Cluster Formulas in Ti-Al(-Nb) Phases

**Figure 2:**
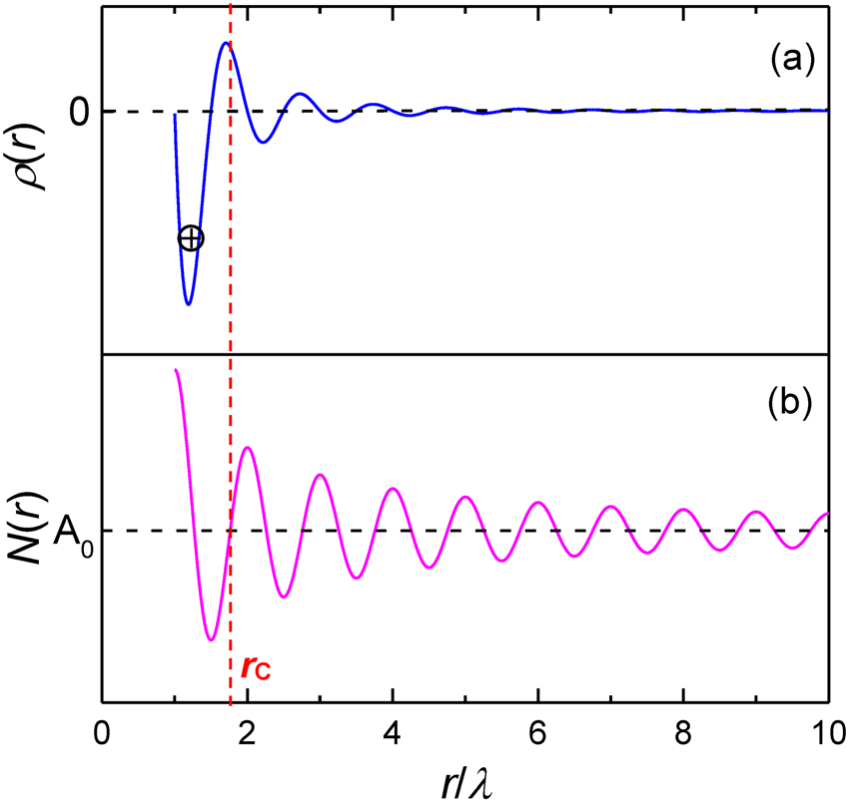
Idealized electron-density oscillations *ρ*(*r*) (**a**) and radial distribution function *N*(*r*) of electrons (**b**).

The establishment of the cluster-plus-glue-atom model was based on the periodic electron wave function in the form of Friedel oscillation, which was used to describe the electron-structure interaction for the phase stabilization, *i*.*e*., the influence of electronic system on the static strucutre^41,42^. The Friedel oscillation theory is generally expressed with a damped cosine-function^36,43^,

1 $$\rho (r)={\rm{A}}[\cos \,(2{k}_{F}\cdot r+\theta )]/{r}^{3}=-\,{\rm{A}}\,[\sin \,(2{k}_{F}\cdot r)]/{r}^{3}(\theta =\pi /2),$$

where *k*_F_ is the Fermi vector, A is the amplitude, *θ* is the phase shift, and the wavelength *λ*_Fr_ of the function is equal to *λ*_Fr_ = 2π/2*k*_F_ = π/*k*_F_. It describes the distribution of electron density around any ion, which itself is caused by the screening behavior of electron cloud^44,45^. It needs to be pointed out that at a short- or medium-range distance, the phase shift *θ* is close to π/2. Thus, the oscillation turns into a sine-function of −A[sin(2*k*_F_ ∙ *r*)]/*r*^3^ ^46–48^, as shown in Fig. 2(a). In such oscillated case, the ions (atoms) tend to occupy the troughs of this function, as a result of the formation of CSROs. Then, the cluster-plus-glue-atom model was introduced into the oscillation to develop a cluster-resonance structural model^19,20^, in which the product of the total number of atoms in a cluster structural unit (*Z*) and the effective electrons per atom (*e/a*) is constant for the ideal metallic glass (*Z* · (*e/a*) = constant). That is to say, the *Z* value is associated with the *e/a* from the viewpoint of CSROs. Recently, the cluster formulas of [cluster](glue atom)_*x*_ for complex metallic alloys, including Ni-based superalloys^49^, high-entropy alloys^22,31^, and Cu-Zn solid-solution alloys^50^, have been determined by the calculated total atom number *Z* values in specific cluster structural units. In the following, the cluster structural units, containing two parts of cluster and glue atoms, in the present Ti-Al(-Nb) phases will be discussed separately.

### Clusters

In a crystalline structure, the cluster is the nearest-neighbor coordination polyhedron to represent the strongest CSRO. It is easy to determine the clusters in FCC, HCP, and BCC solid-solution structures, being the CN12 cuboctahedron (CN: coordination number), CN12 twined cuboctahedron, and CN14 rhombic dodecahedron, respectively. When Al atoms are added into these solid solutions, Al interacts with the base Ti strongly due to the large Δ*H*_Ti-Al_ = −30 kJ/mol^29^. Thus, the typical clusters in *α*-Ti, *ω*-Ti, and *β*-Ti solid solutions are CN12 [Al-Ti_6_Ti_6_], CN14 [Al-Ti_2_Ti_12_], and CN14 [Al-Ti_8_Ti_6_], respectively. For the ordered superstructures, *α*_2_-Ti_3_Al, *γ*-TiAl, and B2-Ti(Al,Nb) phases, the clusters still inherit the similar polyhedrons with the same CN from their parent solid solutions, such as [Al-Ti_6_Ti_6_] in *α*_2_-Ti_3_Al (as seen in Supplementary Table S1). For the *O*-Ti_2_AlNb, there exists a serious separation in several nearest neighbors (Table S1) due to the relatively-weaker interaction between Al-Nb (Δ*H*_Nb-Al_ = −18 kJ/mol) than Al-Ti. According to the determination of the nearest-neighbor cluster distance containing multiple shells^51^, the CN12 cluster [Al-Ti_4_Ti_2_Ti_2_Nb_2_Nb_2_] = [Al-Ti_8_Nb_4_] in *O*-Ti_2_AlNb is then obtained. All these clusters and their diagrams, also including CN12 [Al-Al_4_Ti_8_] in *γ*-TiAl and CN14 [Al-Ti_8_(Al,Nb)_6_] in B2-Ti(Al,Nb), are shown in Table S1.

### Glue atoms

Glue atoms are used to fill in the interstitial sites of cluster packing to balance the atomic density, which is essential in the cluster structural unit. More importantly, the atomic distribution of solutes in glue atom sites will dominate whether the alloy structure is a solid solution or an intermetallic compound. Since the atom number of the cluster part is fixed in these phases, the glue atom number can be determined by the total atom number *Z* in the cluster structural unit, which could be obtained from the Friedel oscillation theory^52^. According to Eq. 1, the total electron number *N* in any sphere with a radius *r* can be obtained by the integration of *ρ*(*r*) from the zero point to *r*:

2 $${N}({r})=-\,4\pi A\,{\int }_{0}^{{r}}\frac{\sin (2{{k}}_{F}\cdot {r})}{{r}}\mathrm{dr}.$$

As shown in Fig. 2(b), *N*(*r*) is also a periodic oscillation function with a wavelength of *λ*_Fr_ and converges to A_0_ at infinity. Since the cluster structural unit involves the strongest interactions (cluster) and the weakest ones (glue atoms), it is regarded to be neutral ideally and the electron number in the cluster structural unit with a spherical radius of *r*_C_ should be equal to A_0_ at infinity. Letting *N*(*r*) = A_0_, one of the solutions of the equation is *r* = 1.764*λ*_Fr_, which covers the minimum and maximum values, *i*.*e*., the first trough and peak in *ρ*(*r*). It is then set to be equal to *r*_C_, *i*.*e*., *r*_C_ = 1.764*λ*_Fr_. Supposing that the atomic density in the cluster structural unit *ρ*_C_ is equal to the average atomic density *ρ*_a_ derived from the unit cell of phase structure, Eq. 3 can be established:

3 $${\rho }_{C}=\frac{Z}{\frac{4}{3}\pi {r}_{C}^{3}}=\frac{{Z}_{0}}{V}={\rho }_{{\rm{a}}},$$

where *Z* is the atom number in the cluster structural unit with a radius *r*_C_, and *Z*_0_ is the atom number in a unit cell with a volume *V*. Putting *r*_C_ = 1.764*λ*_Fr_ into Eqs 3 and 4 can be deduced to calculate the *Z* value, *i*.*e*.,

4 $$Z={\rho }_{{\rm{a}}}(4/3){\rm{\pi }}{r}_{C}^{3}=(4/3){1.764}^{3}\cdot {{\rm{\pi }}}^{4}{\rho }_{{\rm{a}}}\cdot {k}_{F}^{-3}=712.908{\rho }_{{\rm{a}}}\cdot {k}_{F}^{-3},$$

in which the Fermi radius *k*_F_^41^ is expressed with

5 $${k}_{{\rm{F}}}={[3{{\rm{\pi }}}^{2}{\rho }_{{\rm{a}}}\cdot (e/a)]}^{1/3}.$$

In these Ti-Al(-Nb) phases, the ordered B2-Ti(Al,Nb) and *α*_2_-Ti_3_Al are well-known as electron compounds with an *e/a* (effective electrons per atom) value of 1.5, satisfying the Hume-Rothery rule, although both of them have a wide composition range^53^. Take the B2-Ti(Al,Nb) phase for an instance, the *k*_F_ was calculated according to Eq. 5, being *k*_F_ = [3π^2^*ρ*_a_ ∙ (*e/a*)]^1/3^ = 13.60 nm^−1^ with *ρ*_a_ = 2/*a*^3^ = 56.68 nm^−3^. It was then put into Eq. 4 to calculate the *Z* value in the cluster structural unit, as a result of *Z*_B2_ = 16.05. Thus, the glue atom number is 1.05, very close to the integer of 1.0, since the atom number in the cluster [Al-Ti_8_(Al,Nb)_6_] is 15. Thereof, the cluster composition formula of B2-Ti(Al,Nb), derived from the cluster structural unit, is finally determined as [Al-Ti_8_(Al,Nb)_6_](Al,Nb)_1_ (=Ti(Al,Nb)). All the parameters involved in the calculation of *Z* values are listed in Table 1. Similarly, for *α*_2_-Ti_3_Al, the cluster formula is determined as [Al-Ti_6_Ti_6_]Al_3_ (=Ti_12_Al_4_ = Ti_3_Al) with the calculated *Z* = 16.05, in which the type of glue atoms is dependent on the element atoms occupied into the second nearest neighbor (Table S1).

**Table 1:** Crystalline structures, cluster formulas, the total atom number *Z* of cluster structural unit in Ti-Al(-Nb) phases, in which the Fermi vector *k*_F_ and atomic density *ρ*_a_ are also involved.

Phase	Structure	Cluster formula	Composition (at. %)	*Z*	*k*_F_ (nm^−1^)	*ρ*_a_ (nm^−3^)
*α*-Ti	*hP*-Mg	[Al-Ti_6_Ti_6_]Ti_3_	Ti (Al-doped)	16.01	13.62	56.68
*α* (limit Al)	*hP*-Mg	[Al-Ti_6_Ti_6_]AlTi_2_	Ti_87.5_Al_12.5_	—	—	—
*α*_2_ (Ti_3_Al)	*hP*-Ni_3_Sn (D0_19_)	[Al-Ti_6_Ti_6_]Al_3_	Ti_75_Al_25_	16.05	13.80	59.50
*α*_2_ (Ti_70_Al_23_Nb_7_)	*hP*-Ni_3_Sn (D0_19_)	[Al-Ti_6_Ti_5_Nb_1_]Al_3_	Ti_68.75_Al_25_Nb_6.25_	—	—	—
*γ* (TiAl)	*tP*-AuCu (L1_0_)	[Al-Al_4_Ti_8_]Al_3_	Ti_50_Al_50_	15.89	14.02	61.38
*O* (Ti_2_AlNb)	*oC*-HgNa	[Al-Ti_4_Ti_2_Ti_2_Nb_2_Nb_2_]Al_3_ = [Al-Ti_8_Nb_4_]Al_3_	Ti_50_Al_25_Nb_25_	15.74	13.78	56.73
B2 (Ti(Al,Nb))	*cP*-CsCl	[Al-Ti_8_(Al,Nb]_6_](Al,Nb)	Ti_50_(Al,Nb)_50_	16.05	13.60	56.68
*β*-Ti (>882 °C)	*cI*-W	[Al-Ti_8_Ti_6_]Ti	Ti (Al-doped)	16.26	13.42	55.09
*ω*-Ti	*hP*-AlB_2_	[Al-Ti_2_Ti_12_]Ti	Ti (Al-doped)	16.27	13.65	58.05

Unlike B2 and *α*_2_, the ordered *γ*-TiAl and *O*-Ti_2_AlNb phases don’t exhibit definite *e/a* values. However, the phase stabilization is still dominated by the universal Fermi sphere-Brillouin zone (FS-BZ) interaction mechanism, *i*.*e*., 2*k*_F_ = *K*_P_^53,54^, in which *K*_P_ is the width of Brillouin zone with *K*_P_ = 2π/*d* (*d*: interplanar spacing). Thus, we can calculate the *k*_F_ through *K*_P_ that can be obtained according to the strong diffraction planes. Actually, the electronic states near the Fermi level were often found to be perturbed by the resonance of electrons with different sets of lattice planes^55^. So a weight coefficient *ω*_*i*_ is introduced here to consider the co-action of several principal diffraction planes {hkl}, which is defined by the intensity ratio of the principal peaks. Thus, the *K*_P_ is expressed with the equation:

6 $${{K}}_{P}=\sum _{{i}=1}^{{n}}{{\omega }}_{{i}}\cdot \frac{2\pi }{{d}}.$$

Take the *O*-Ti_2_AlNb phase for an example, there exist four strong diffraction planes of {040}, {002}, {221}, and {041} near the Fermi level, from which the *K*_P_ was calculated to be *K*_P_ = 27.56 nm^−1^ = 2*k*_F_. Putting it into Eq. 4, the calculated *Z* value is 15.74, close to the integer of 16. So, the cluster formula of the *O*-Ti_2_AlNb phase is determined as [Al-Ti_8_Nb_4_]Al_3_ (=Ti_8_Al_4_Nb_4_ = Ti_2_AlNb). Similarly, the cluster formula of *γ*-TiAl is [Al-Al_4_Ti_8_]Al_3_ (=Ti_8_Al_8_ = TiAl). Using this equation, the *Z* value in the cluster structural unit of the B2 phase is also calculated, as *Z* = 16.27, which is consistent with the value of *Z* = 16.05 with the Eq. 5.

The *e/a*-scaled Hume-Rothery rule was also validated in solid-solution alloys, especially when the amount of solute elements reaches the solubility limit. It is applicable in Ti solid-solution alloys due to a fair amount of alloying elements for solution strengthening, as exemplified by the fact that the Al content was generally up to 5 ~ 6 wt.% (8.5 ~ 10.2 at.%)^56^, very close to solubility limit of 12.5 at. % Al. The *K*_P_ values of BCC *β*-Ti (high-temperature phase), hexagonal *ω*-Ti (intermediate phase) and HCP *α*-Ti (low-temperature phase) were calculated according to their principal diffraction planes. Then the *Z* values in their cluster structural units are obtained, being *Z*_*β*_ = 16.26, *Z*_*ω*_ = 16.27, and *Z*_*α*_ = 16.01, respectively, which are also very close to the integer of 16. Thus, the cluster formulas of *β*-Ti, *ω*-Ti, and *α*-Ti are determined to [Al-Ti_8_Ti_6_]Ti_1_, [Al-Ti_2_ Ti_12_]Ti_1_, and [Al-Ti_6_Ti_6_]Ti_3_, respectively.

### Relationships of cluster structural units (cluster formulas) among Ti-Al(-Nb) phases

**Figure 3:**
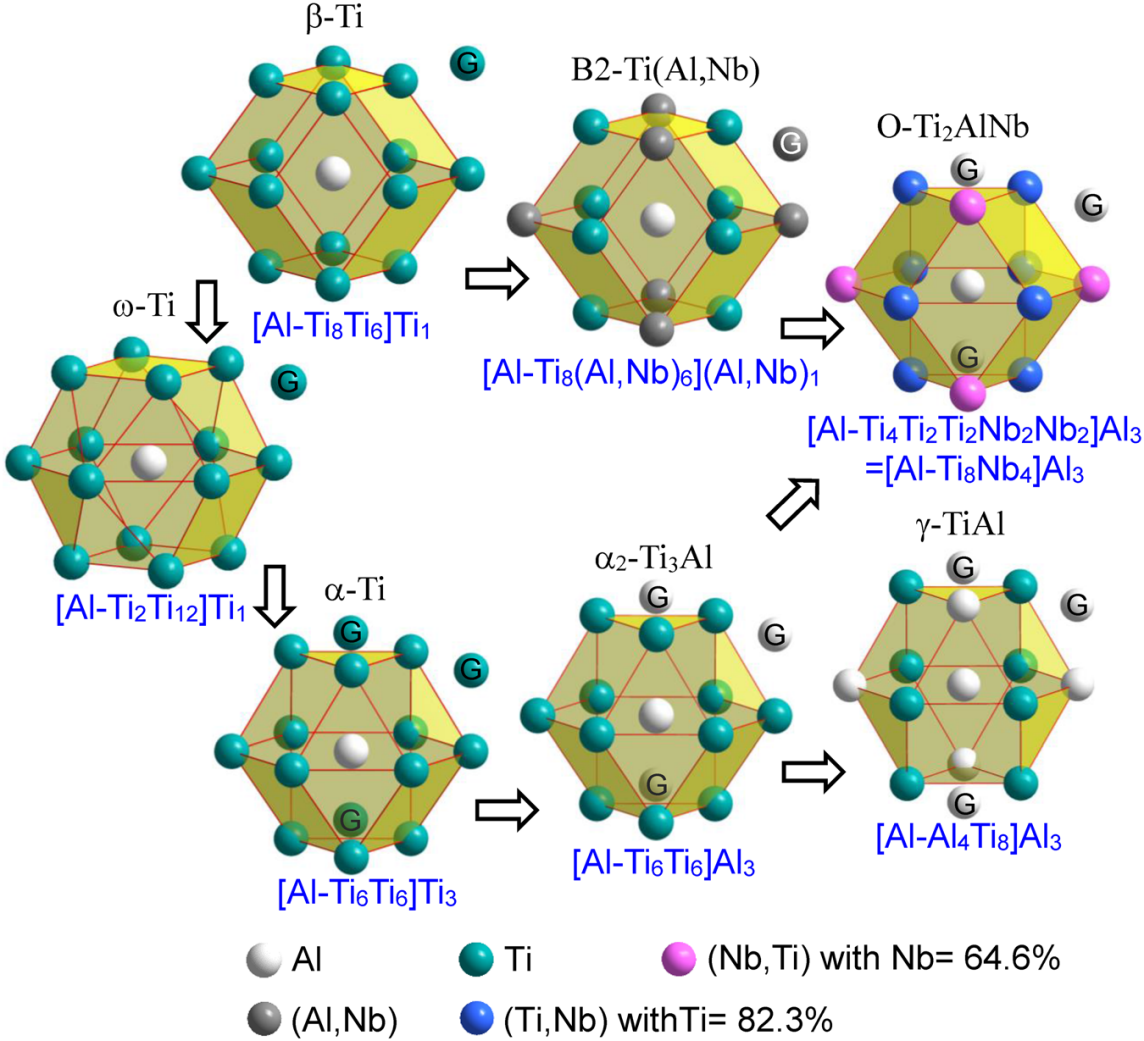
Cluster structural units centered by Al atom and their relationships in Ti-Al(-Nb) phases. The atoms outside the cluster (the yellow enclosed polyhedron), marked with ‘G’, are glue atoms, in which atom spheres with different colors represent different alloying elements.

From above, it can be concluded that all these cluster formulas of Ti-Al(-Nb) phases contain a constant total atom number of *Z* = 16 in their structural units, which could be favorable to the phase transformation. There exist close relationships among these phases from the viewpoint of local CSROs (cluster structural units), as shown in Fig. 3. When a small amount of Al is added into the base Ti, Al must be surrounded by Ti atoms due to the Al-Ti strong interaction to form [Al-Ti_12_] clusters in *α*-Ti solid solution. Increasing the Al content, Al can enter the glue atom sites to replace Ti, in which one of the cluster formula [Al-Ti_6_Ti_6_](AlTi_2_) with one Al in glue atom sites corresponds to the upper limit of the solid solubility (12.5 at.% Al). Once more Al atoms appear at the glue sites, the strong interaction between Ti and Al will contribute to the precipitation of *α*_2_-Ti_3_Al from its parent *α*-Ti solid solution, as evidenced by many experiments^57,58^. Such kind of phase transformation is easy to occur, since it needs to adjust atoms in local short range alone. Thus, the [Al-Ti_6_Ti_6_]Al_3_ represents the local structural unit of *α*_2_-Ti_3_Al, in which the cluster part is same as that of *α*-Ti. Further increasing the Al content, Al atoms enter into the cluster shell gradually to form [Al-Al_4_Ti_8_]Al_3_, the cluster formula of *γ*-TiAl phase, in which the phase structure changes from the ordered HCP to a tetragonal structure due to the lattice distortion. Thereof, the adjustment of local cluster structural units can induce the phase transformation, which has been identified in Al-Fe-Co-Ni-Cr alloys by neutron scattering experiments^22,31^. When Nb is gradually added into these Ti-Al compounds to form *O*-Ti_2_AlNb and B2-Ti(Al,Nb) phases, Nb atoms tend to enter into the external cluster shell to replace the base Ti due to the relatively-weaker interaction of Al-Nb than that of Al-Ti. One of the substitutions can form the formula of [Al-Ti_6_Ti_5_Nb_1_]Al_3_ (Ti_70_Al_23_Nb_7_, at.%), which corresponds to the maximum content of Nb in *α*_2_-Ti_3_Al phase^59^. Moreover, the excess Nb substitution for Ti could lead to a large lattice distortion, as a result of the formation of other compounds, such as [Al-Ti_8_Nb_4_]Al_3_ for the *O*-Ti_2_AlNb phase. Apparently, the cluster structural unit of *O*-Ti_2_AlNb phase ([Al-Ti_8_Nb_4_]Al_3_) is between *α*_2_-Ti_3_Al ([Al-Ti_6_Ti_6_]Al_3_) and B2-Ti(Al,Nb) ([Al-Ti_8_(Al,Nb)_6_](Al,Nb)_1_) from the viewpoint of local atomic distribution, since the two atoms marked with ‘G’ in Fig. 3 shift from the glue sites (in *α*_2_ and *O*) to the cluster-shell sites (B2) gradually. Compared with *β*-Ti, a similar CN14 cluster still keeps in the intermediate *ω*-Ti phase, but a slight distortion occurs in the cluster. Therefore, it is due to the change of local cluster structural units to induce the phase transformation of crystal structures, which is attributed to the amounts of alloying elements of Al and Nb.

## Cluster Formulas of Industrial Ti Alloys

Since the cluster formula approach describes well the phase compositions of Ti solid solutions and Ti-Al intermetallic compounds, it can certainly reveal the composition rules in complex industrial Ti alloys developed on the basis of these phases. In fact, in multi-component Cu solid-solution alloys, there indeed exists a simple composition rule behind seemingly complex chemistries of Cu alloys with the guide of the cluster-formula approach, which offers a fundamental and practical method towards composition interpretations of industrial alloys^50^. Multiple alloying elements, such as Al, Sn, Mo, Nb, Ta, V, Cr, Fe, etc., are usually co-added into industrial Ti alloys, regardless of near *α*-Ti, *α*_2_-Ti_3_Al based, *O*-Ti_2_AlNb based, *γ*-TiAl based, and *β*-Ti alloys. The variation among them is that the amounts of alloying elements are different in order to form various phase structures and to satisfy the service performance. For example, Al is a strong *α* stabilizer, while the β stabilizers (Mo, Nb, Ta, V, Cr, Fe, etc.) contribute to the formation of *β* and B2 phases. In the following, we will interpret the composition rules of different kinds of Ti alloys with the cluster formula approach.

### High-temperature near *α*-Ti alloys

The high-temperature near *α*-Ti alloys have the prominent specific strength and creep-resistance properties for aerospace applications at 600 °C^5^. These alloys contain the primary alloying element Al with a content of 5 ~ 7 wt. %, and a small amount of β-stabilizing elements of Mo, Nb, Ta, as well as some neutral elements of Zr and Sn^4,60^. Since they exhibit the HCP-*α* solid-solution structure, the cluster formula [Al-Ti_12_]Ti_3_ for the *α*-Ti phase is applied to interpret the complex compositions of this kind of alloys. It is noted that Al atoms occupy the cluster center preferentially and then enter the glue atom sites according to the cluster formulas of *α*-Ti and *α*_2_-Ti_3_Al. The Zr element will substitute for the base Ti in the cluster shell due to the Δ*H*_Ti-Zr_ with a value of zero. Other minor β-stabilizing elements M, like Mo, Nb, and Si, etc., are also set into the glue atom sites due to the weaker interactions of M-Ti than Al-Ti. Thus, on the premise of fixing the atom numbers of Ti and Zr on the cluster shell as CN12, the cluster formulas of the typical high performance near *α*-Ti alloys were analyzed and listed in Table 2. For instance, the Ti-6Al-2.75Sn-4Zr-0.4Mo-0.45Si (Ti-1100, wt.%) alloy (Ti_85.26_Al_10.52_Sn_1.10_Zr_2.07_Mo_0.20_Si_0.76_, at. %)^58^ could be expressed with the cluster formula of [Al-Ti_11.67_Zr_0.33_](Ti_2_Al_0.68_Sn_0.18_Mo_0.03_Si_0.12_). More importantly, the total atom number *Z* of the cluster formula (the cluster structural unit) is 16.01, very close to *Z* = 16. A similar rule also lies in other typical alloys, such as Ti-5.8Al-4Sn-3.5Zr-0.7Nb-0.5Mo-0.35Si-0.06 C (IMI834, wt.%)^9^ and Ti-6Al-2.8Sn-4Zr-0.5Mo-0.4Si-0.1Y (Ti600, wt.%)^61^ alloys (listed in Table 2). Thereof, the general cluster formula of the industrial near *α*-Ti alloys could be expressed by the cluster formula of [Al-(Ti,Zr)_12_](Ti_2_(Al,M)_1_) with *Z* = 16, where M represents the β-stabilizing elements.

**Table 2:** Cluster formulas and their total atom number *Z* of industrial Ti alloys, in which alloy compositions (in wt.% or at.%), alloy types, phase constitution, and the service temperatures are also listed.

Alloys	Phase	Composition	Cluster formulas	*Z*	Refs	Temperature (°C)
Near α-Ti alloys	α + minor β	Ti-5.8Al-4Sn-3.5Zr-0.7Nb-0.5Mo-0.35Si-0.06 C (wt.%) (IMI834)	Al-Ti_11.7_Zr_0.3_] (Ti_2_Al_0.66_Sn_0.26_Mo_0.04_Nb_0.06_Si_0.1_)	16.11	^9^	600
		Ti-6Al-2.75Sn-4Zr-0.4Mo-0.45Si (wt.%) (Ti1100)	[Al-Ti_11.67_Zr_0.33_] (Ti_2_Al_0.68_Sn_0.18_Mo_0.03_Si_0.12_)	16.01	^58^	
		Ti-6Al-2.8Sn-4Zr-0.5Mo-0.4Si- 0.1Y (wt.%) (Ti600)	[Al-Ti_11.67_Zr_0.33_] (Ti_2_Al_0.69_Sn_0.18_Mo_0.04_Si_0.11_)	16.01	^61^	
		Ti-6.2Al-2Sn-3.6Zr-0.7Mo-0.15Si-5W (wt.%) (BT36)	[Al-Ti_13.69_Zr_0.31_]- (Al_0.83_Sn_0.13_Mo_0.06_Si_0.04_W_0.22_)	16.28	^60^	
Ti_3_Al-based alloys	(α_2_ + B2)/(α_2_ + B2 + O)	Ti-23Al-7Nb (Ti_3_Al-Nb) (at.%)	[Al-Ti_10.91_Nb_1.09_]Al_2.58_	15.58	^59^	650–700
		Ti-25Al-8Nb-2Mo-2Ta (at.%)	[Al-Ti_10.08_Mo_0.32_Nb_1.28_Ta_0.32_]Al_3_	16.00	^2^	
		Ti-24Al-11Nb (at.%)	[Al-Ti_10.26_Nb_1.74_]Al_2.79_	15.79	^63^	
		Ti-24Al-12.5 Nb (at.%)	[Al-Ti_10.03_Nb_1.97_]Al_2.79_	15.79	^65^	
		Ti-24Al-17Nb (at.%)	[Al-Ti_9.32_Nb_2.68_]Al_2.79_	15.79	^66^	
		Ti-25Al-10Nb-3V-1Mo (at.%)	[Al-Ti_9.76_Mo_0.16_Nb_1.6_V_0.48_]Al_3_	16.00	^11^	
Ti_2_AlNb- based alloys	O + B2 + minor α_2_	Ti-25Al-25Nb (at.%)	[Al-Ti_8_Nb_4_]Al_3_	16.00	^68^	650–750
		Ti-22Al-(23~27)Nb (at.%)	[Al-Ti_8.15_Nb_3.85_]Al_2.38_	15.38	^67^	
TiAl-based alloys	γ + α_2_ + minor β/B2	Ti-44Al-4~6Nb-1Mo (at.%)	[Al-Al_3.04_Ti_8_Nb_0.8_Mo_0.16_]Al_3_	16.00	^72^	750–950
		Ti-43Al-4Nb-1.5Mo-0.1B-0.5 C (at.%)	[Al-Al_3.29_Ti_8_Nb_0.62_Mo_0.23_]Al_2.4_	15.40	^73^	
		Ti-43Al-9V-0.2Y (at.%)	[Al-Al_2.46_Ti_8_V_1.51_Y_0.03_]Al_3.74_	16.74	^74^	
		Ti-46Al-2Cr-2Nb (at.%)	[Al-Al_3.36_Cr_0.32_Ti_8_Nb_0.32_]Al_3_	16.00	^75^	
		Ti-45Al-4Nb-2Mo (at.%)	[Al-Al_3.02_Ti_8_Mo_0.33_Nb_0.65_]Al_3.33_	16.33	^76^	
		Ti-47Al-2W-0.2Si (at.%)	[Al-Al_3.65_Ti_8_W_0.31_Si_0.03_]Al_2.75_	15.75	^77^	
β-Ti alloys	β + α	Ti-15Mo-5Zr-3Al (wt.%)	[(Al_0.91_Mo_0.09_)-Ti_14_]Mo_1.19_	16.19	^56^	<500
		Ti-3Al-5Mo-4.5 V (wt.%) (VT16)	[(Al_0.91_Mo_0.09_)-Ti_14_](Mo_0.34_V_0.72_)	16.06	^78^	
		Ti-5Al-2Sn-2Cr-4Mo-4Zr-1Fe (wt.%) (β-CEZ)	[Al-Ti_13.65_Zr_0.35_] (Al_0.48_Sn_0.13_Mo_0.33_Fe_0.14_Cr_0.31_)	16.39	^56^	
		Ti-5Al-2Sn-2Zr-4Mo-4Cr (wt.%) (Ti-17)	[Al-Ti_13.83_Zr_0.17_] (Al_0.48_Sn_0.13_Mo_0.33_Cr_0.61_)	16.56	^56^	
		Ti-7Mo-3Nb-3Cr-3Al (wt.%) (Ti-7333)	[Al_0.91_Cr_0.09_-Ti_14_] (Mo_0.6_Nb_0.26_Cr_0.38_)	16.24	^79^	
		Ti-4.5Al-6.5Mo-2Cr-2.6Nb-2Zr- 1Sn (wt.%) (TB17)	[Al-Ti_13.82_Zr_0.18_] (Al_0.36_Sn_0.07_Mo_0.55_Nb_0.23_Cr_0.31_)	16.51	^80^	
		Ti-5Al-5Mo-5V-1Fe-1Cr (wt.%) (BT22)	[Al-Ti_14_] (Al_0.5_Mo_0.42_Fe_0.14_V_0.79_Cr_0.16_)	17.01	^2^	
	β/(β + α)	Ti-12Mo (wt.%)	[Mo-Ti_14_]Ti_0.7_	15.70	^81^	
		Ti-5.2Mo-6.5Sn-10Zr-10.2 Nb (wt.%)	[(Mo_0.5_Sn_0.5_)-Ti_13_Zr_1_]Nb	16.00	^33^	
		Ti-12Mo-6Zr-2Fe (wt.%) (TMZF)	[(Mo_0.71_Fe_0.29_)-Ti_13.47_Zr_0.53_]Mo_0.3_	15.30	^2^	
		Ti-10V-2Fe-3Al (wt.%) (Ti-1023)	[Al_0.88_-Ti_14_](Fe_0.28_V_1.55_)	16.71	^56^	
		Ti-15Mo-3Al-2.7Nb-0.25Si (wt.%) (β21S)	[Al_0.94_-Ti_14_](Mo_1.33_Nb_0.25_Si_0.08_)	16.59	^56^	
		Ti-1Al-8V-5Fe (wt.%) (1-8-5)	[(Al_0.29_Fe_0.7_)-Ti_14_]V_1.22_	16.21	^56^	

### Ti_3_Al-based intermetallic alloys

In order to elevate the service temperature of *α*-Ti alloys up to 700 °C, Ti_3_Al-based intermetallic alloys were then developed, possessing higher specific strengths and good oxidation-resistance^62^. However, an obvious defect of this kind of alloys is their poor tensile ductility and toughness at room temperature due to the brittle Ti_3_Al structure. Thus, some β-stabilizing elements, like Nb and Mo, were often added into these alloys to improve their ductility induced by the formation of a small amount of *β*/B2 phases^63^. According to the cluster formula [Al-Ti_12_]Al_3_ of *α*_2_-Ti_3_Al phase, the β-stabilizers M will substitute for the base Ti on the cluster shell to analyze the compositions of this kind of alloys. Indeed, it has been identified experimentally that the β-stabilizing elements (V, Cr, Mn, Zr, Nb, Mo, and Ta) strongly favor to enter the Ti sites in Ti_3_Al^64^. Take the Ti-25Al-12.5 Nb (at.%) alloy with a low Nb content for instance^65^, the composition is well interpreted by the cluster formula of [Al-(Ti_10_Nb_2_)]Al_3_ with two Nb atoms substitution for Ti and with *Z* = 16. For the Ti-24Al-17Nb (at.%) alloy with a high Nb content^66^, the cluster formula is expressed with [Al-(Ti_9.32_Nb_2.68_)]Al_2.79_, containing approximate three Nb atoms and with *Z* = 15.79. All the cluster formulas of typical Ti_3_Al-based alloys are listed in Table 2, from which it can be derived that the total atom number *Z* values of the cluster formulas are all very close to *Z* = 16 with a general formula of [Al-(Ti_12-*y*_M_*y*_)]Al_3_ (*y* = 1 ~ 3). In addition, with increasing the Nb content, B2 and *O*-Ti_2_AlNb phases will successively precipitate from the *α*_2_-Ti_3_Al matrix.

### Ti_2_AlNb based alloys

Further increasing the Nb content in above Ti_3_Al-based alloys, another kind of intermetallic alloys based on *O*-Ti_2_AlNb was obtained with a Nb content being about 23 ~ 27 at. %^67,68^, which exhibit a better ductility and toughness than the former. For the typical Ti-25Al-25Nb alloy, it shows the composition ratio of the *O*-Ti_2_AlNb phase, and the cluster formula is [Al-Ti_8_Nb_4_]Al_3_ with *Z* = 16. For Ti-22Al-(23 ~ 27)Nb (at.%) alloys with a relatively-low content of Al, the cluster formula is [Al-Ti_8.15_Nb_3.85_]Al_2.38_ with *Z* = 15.38, as listed in Table 2. It is noted that the number of Nb atoms in this kind of alloys is close to 4.

### *γ***-**TiAl based alloys

The TiAl-based alloys are generally used at a much higher temperature above 800 °C, and are considered as the promising high-temperature materials to replace Ni-based superalloys because of their lower density, better oxidation resistance, and attractive mechanical properties at elevated temperatures^6,69^. They exhibit a lamellar microstructure consisting of *γ*-TiAl, *α*_2_-Ti_3_Al (>30%) and minor B2/*β* phases due to the addition of β-stabilizing elements M (M = Nb, V, Cr, or Mo) for improvement of room-temperature ductility and toughness^69–71^. Thus, the cluster composition formulas of these alloys could not be determined alone based on the [Al-Al_4_Ti_8_]Al_3_ of *γ*-TiAl since there exists a certain amount of *α*_2_-Ti_3_Al ([Al-Ti_6_Ti_6_]Al_3_). For simplicity, the β-stabilizing elements M are arbitrarily set to replace the Al atoms in the cluster shell of [Al-Al_4_Ti_8_], in accordance with the M substitution for Ti in the cluster [Al-Ti_12_] of Ti_3_Al phase. Finally, the cluster formula of [Al-(Al,M)_4_Ti_8_]Al_*x*_ is taken to analyze the compositions of TiAl-based alloys, as listed in Table 2. For example, Ti-44Al-(4 ~ 6)Nb-1Mo (at.%) alloys^72^ can be expressed by an average cluster formula of [Al-Al_3.04_Ti_8_Nb_0.8_Mo_0.16_]Al_3_ with *Z* = 15.69 ~ 16.33, close to the integer of 16.

### *β*-Ti alloys

*β*-Ti alloys have been applied widely into both biomedicine and aviation fields due to the low Young’s modulus and good biocompatibility, high strength (above 1300 MPa) and excellent fatigue/crack-propagation behavior^4^. These alloys are always consisted of primary *β* phase plus a small amount of *α* and *ω* phases, since a much more amount of β stabilizers of Mo, V and Cr were added besides a relatively small amount of Al, Zr and Sn. Therefore, the cluster formula of [(Al,M)-Ti_14_]Ti for *β*-Ti is applied to understand the complex compositions of this kind of alloys. The cluster center is preferentially occupied by the solute having a large negative Δ*H* with Ti, such as Al, Fe, or Mo. Zr still substitutes for the base Ti on the cluster shell due to the zero Δ*H*_Ti-Zr_. Other alloying elements, like Nb, Ta and V, etc., are set into the glue atom sites due to the weaker interactions with Ti. It is found from Table 2 that the *Z* values of near *β*-Ti alloys are about 15.5 ~ 16.5. For instance, the [(Mo_0.5_Sn_0.5_)-(Ti_13_Zr_1_)]Nb_1_ alloy with a low Young’s modulus of *E* = 48 GPa, which is obtained by the cluster formula with *Z* = 16, *i*.*e*., one CN14 cluster matching with one glue atom^33^. Another alloy of Ti-15Mo-5Zr-3Al (wt.%)^56^ could be explained with the cluster formula [(Al_0.91_Mo_0.09_)-Ti_14_]Mo_1.19_ with *Z* = 16.19. Thus, the general cluster formula of *β*-Ti alloys could be expressed by the formula of [(Al,M1)-(Ti,Zr)_14_](M2)_1_ with *Z* = 16, in which M1 and M2 represent one type of β-stabilizing elements having strong interactions with Ti and the other type having weak interactions with Ti, respectively. However, for the Al-contained *β*-Ti industrial alloys, it must add a much greater amount of β stabilizers to compensate the negative effect of the α-stabilizer Al. Therefore, the total atom number *Z* of some high-strength/toughness *β*-Ti alloys might be higher than 16, which is attributed to the increase of glue atoms for the occupation of β stabilizers. For instance, the cluster formula of the Ti-15Mo-3Al-2.7Nb-0.25Si (β21 S, wt.%) alloy is described with the form of [Al_0.94_-Ti_14_](Mo_1.33_Nb_0.25_Si_0.08_) with *Z* = 16.59.

## Conclusions

The cluster-plus-glue-atom model for the characterization of CSROs was introduced to explore the relationships among the local atomic distributions in *α*-Ti, *ω*-Ti, *β*-Ti, *α*_2_-Ti_3_Al, *γ*-TiAl, *O-*Ti_2_AlNb, and B2-Ti(Al,Nb) phases. Based on this approach, the composition rules of the multi-component Ti solid-solution and intermetallic alloys were investigated. The main conclusions are described as follows:

Specific cluster structural units for Ti-Al(-Nb) phases are determined according to the Friedel oscillation theory for the electron-structure stabilization. It is due to the change of cluster structural units that induces the phase transformation, which is attributed to the amounts of alloying elements of Al and Nb. The total atom number *Z* in a cluster structural unit is closely related to the Fermi vector *k*_F_, which can be calculated by the equation of *Z* = (4/3)∙1.764^3^∙π^4^*ρ*_a_∙*k*_F_^−3^ = 712.908*ρ*_a_ ∙ *k*_F_^−3^. It is found that the values of *Z* in all these phases are very close to *Z* = 16. Since the cluster parts are taken as the nearest-neighbor coordination polyhedron in each phase, the specific cluster formulas for these phases are finally determined as: [Al-Ti_12_]Ti_3_ for *α*-Ti, [Al-Ti_12_]Al_3_ for *α*_2_-Ti_3_Al, [Al-Al_4_Ti_8_]Al_3_ for *γ*-TiAl, [Al-Ti_8_Nb_4_]Al_3_ for *O*-Ti_2_AlNb, [Al-Ti_8_(Al,Nb)_6_](Al,Nb)_1_ for B2-Ti(Al,Nb), and [Al-Ti_14_]Ti_1_ for *β*-Ti, respectively.

The composition rules of the multi-component Ti solid-solution and intermetallic alloys are achieved in light of the cluster formulas of these phases with *Z* = 16. For the near *α*-Ti solid-solution alloys, their compositions can be well described with the cluster formula [Al-(Ti,Zr)_12_](Ti_2_(Al,M)_1_), where M represents the β-stabilizing elements. For *β*-Ti solid solution alloys, their compositons satisfy the uniform cluster formula of [(Al,M)-(Ti,Zr)_14_](M)_1_. For the intermetallic-based Ti alloys with a high amount of Al for improvement of high-temperature performance, alloy compositions satisfy the cluster formulas of [Al-(Ti,M)_12_]Al_3_ for Ti_3_Al-based alloys and [Al-(Al,M)_4_Ti_8_]Al_3_ for TiAl-based alloys, respectively. Among them, one special formula of [Al-(Ti_8_Nb_4_)]Al_3_ (M = Nb) can interpret the composition of Ti_2_AlNb-based alloys.

## Supplementary information

Dataset 1

## Data Availability

The data used in this article are available from the corresponding author on request.
