# Supplementary material for: Exploration of phase structure evolution induced by alloying elements in Ti alloys via a chemical-short-range-order cluster model
Source: Sci Rep. 2019 Mar 4;9:3404. doi: 10.1038/s41598-019-40302-5 (PMC6399271; doi:10.1038/s41598-019-40302-5)
Supplement: Supplementary file 1 — Dataset 1 [file 41598_2019_40302_MOESM1_ESM.pdf]

Supplementary Information

Exploration of phase structure evolution induced by alloying elements in Ti alloys via a chemical-short-range-order cluster model

Beibei Jiang[1], Qing Wang[1,*], Chuang Dong[1], and Peter K. Liaw[2]

[1] *Key Laboratory for Materials Modification by Laser, Ion and Electron Beams (Ministry of Education), School of Materials Science and Engineering, Dalian University of Technology, Dalian 116024, China*

[2] *Department of Materials Science and Engineering, The University of Tennessee, Knoxville, Tennessee 37996, USA*

[*] *Corresponding author. E-mail address: wangq@dlut.edu.cn (Q. Wang)*

Table S1 Phase structure information of Ti-Al(-Nb) phases, including lattice constants (a, b, c), and the atomic distributions around the center atom in the nearest neighbors that contain atomic distance, atom species and numbers on each neighbor shell.

Phase (center atom)	Distance (nm)	Atom occupation	Atom number	Cluster
α (Ti)<br>a = 0.29504 nm<br>c = 0.46810 nm	1st: 0.28947	Ti	6	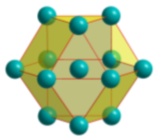<br>[M-Ti_6Ti_6]
	2nd: 0.29504	Ti	6	
	3rd: 0.41333	Ti	6	
	4th: 0.46810	Ti	2	
	5th: 0.50783	Ti	12	
β (Ti)<br>a = 0.33112 nm	1st: 0.28676	Ti	8	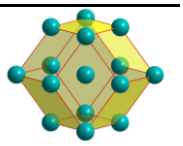<br>[M-Ti_8Ti_6]
	2nd: 0.33112	Ti	6	
	3rd: 0.46827	Ti	12	
	4th: 0.54910	Ti	24	
ω (Ti)<br>a = 0.46000 nm<br>c = 0.28200 nm	1st: 0.28200	Ti	2	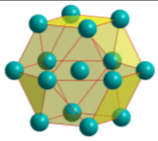<br>[M-Ti_2Ti_{12}]
	2nd: 0.30069	Ti	12	
	3rd: 0.46000	Ti	6	
	4th: 0.49946	Ti	12	
γ-TiAl (Al)<br>a = 0.40010 nm<br>c = 0.40710 nm	1st: 0.28291	Al	4	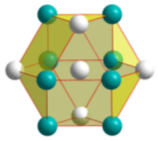<br>[Al-Al_4Ti_8]
	2nd: 0.28540	Ti	8	
	3rd: 0.40010	Al	4	
	4th: 0.40710	Al	2	
	5th: 0.49146	Ti	16	
α_2-Ti_3Al (Al)<br>a = 0.57800 nm<br>c = 0.46470 nm	1st: 0.28586	Ti	6	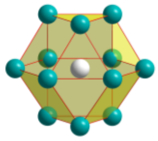<br>[Al-Ti_6Ti_6]
	2nd: 0.28900	Ti	6	
	3rd: 0.40663	Al	6	
	4th: 0.46470	Al	2	
	5th: 0.49909	Ti	12	
O-Ti_2AlNb (Al)<br>a = 0.60893 nm<br>b = 0.95694 nm<br>c = 0.46666 nm	1st: 0.27998	Ti	4	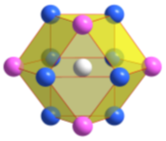<br>[Al-Ti_8Nb_4]
	2nd: 0.28272	Ti	2	
	3rd: 0.28515	Ti	2	
	4th: 0.30239	Nb	2	
	5th: 0.30561	Nb	2	
	6th: 0.39003	Al	2	
	7th: 0.41794	Al	4	
	8th: 0.45206	Nb	1	
	9th: 0.46666	Al	2	
	10th: 0.47855	Nb	4	
B2 (Al)<br>a = 0.32000 nm	1st: 0.28406	Ti	8	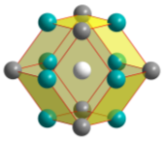<br>[Al-Ti_8Al_6]
	2nd: 0.32800	Al/Nb	6	
	3rd: 0.46286	Al/Nb	12	
	4th: 0.54393	Ti	24	
